# Neuropeptides as Primary Mediators of Brain Circuit Connectivity

**DOI:** 10.3389/fnins.2021.644313

**Published:** 2021-03-11

**Authors:** Mathilde C. C. Guillaumin, Denis Burdakov

**Affiliations:** Department of Health Sciences and Technology, ETH Zürich, Zurich, Switzerland

**Keywords:** hypothalamus, neuropeptides, orexin, hypocretin, arousal, neural circuit

## Abstract

Across sleep and wakefulness, brain function requires inter-neuronal interactions lasting beyond seconds. Yet, most studies of neural circuit connectivity focus on millisecond-scale interactions mediated by the classic fast transmitters, GABA and glutamate. In contrast, neural circuit roles of the largest transmitter family in the brain–the slow-acting peptide transmitters–remain relatively overlooked, or described as “modulatory.” Neuropeptides may efficiently implement sustained neural circuit connectivity, since they are not rapidly removed from the extracellular space, and their prolonged action does not require continuous presynaptic firing. From this perspective, we review actions of evolutionarily-conserved neuropeptides made by brain-wide-projecting hypothalamic neurons, focusing on lateral hypothalamus (LH) neuropeptides essential for stable consciousness: the orexins/hypocretins. Action potential-dependent orexin release inside and outside the hypothalamus evokes slow postsynaptic excitation. This excitation does not arise from modulation of classic neurotransmission, but involves direct action of orexins on their specific G-protein coupled receptors (GPCRs) coupled to ion channels. While millisecond-scale, GABA/glutamate connectivity within the LH may not be strong, re-assessing LH microcircuits from the peptidergic viewpoint is consistent with slow local microcircuits. The sustained actions of neuropeptides on neuronal membrane potential may enable core brain functions, such as temporal integration and the creation of lasting permissive signals that act as “eligibility traces” for context-dependent information routing and plasticity. The slowness of neuropeptides has unique advantages for efficient neuronal processing and feedback control of consciousness.

## Functional Circuit Connectivity, Fast and Slow

In modern neuroscience textbooks, coverage of functional interactions between neurons and their postsynaptic targets remains biased toward fast (millisecond-level on/off) interactions mediated by small-molecule neurotransmitters such as ACh, GABA, and glutamate (Bear et al., 2015). This is presumably due to the enduring influence of the insightful electrophysiological studies of fast neurotransmission by giants of 20th century neuroscience, such as Bernard Katz and John Eccles, which attracted a number of Nobel prizes.

While these fast interactions are undoubtedly one fundamental aspect of brain function, most neurotransmitters in the brain do not operate on these rapid timescales. For example, neuropeptides–which are the largest known class of neurotransmitters (>100)–alter postsynaptic neuronal activity at timescales more similar to typical behaviors and important sensory associations, typically seconds to minutes (Hokfelt et al., 2003; Burdakov, 2004; Salio et al., 2006; Burbach, 2011; Svensson et al., 2018).

These slower actions of neuropeptide transmitters are often described as “modulatory.” This term is not clearly defined, but implies a qualitative difference, or that the main role of the peptides is to change the action of fast neurotransmitters (van den Pol, 2012). However, at the level of neural circuit functional connectivity and control of neuronal spiking, the actions of at least some behaviorally-vital neuropeptides are not conceptually different from fast transmitters: they are simply slower.

The role of neuropeptides in behavioral control, and more specifically, in sleep regulation and vigilance state switching, and their impact on the sleep electroencephalogram has been long known (Steiger and Holsboer, 1997). Instead of reviewing the current knowledge of the role of those peptides in sleep and sedation, we will focus on a few proof-of-concept examples to illustrate their role in neural circuit connectivity–and therefore in brain and behavioral states–beyond the merely modulatory roles which they are often attributed.

As an illustration of this, consider the actions of the prototypical fast transmitter glutamate vs. those of the neuropeptide transmitter orexin/hypocretin (de Lecea et al., 1998; Matsuki and Sakurai, 2008; Schöne et al., 2014). Both are stored in vesicles located in presynaptic axonal terminals, and released upon electrical stimulation of the axons (de Lecea et al., 1998; Torrealba et al., 2003; Schöne and Burdakov, 2012; Schöne et al., 2014). Both bind to specific postsynaptic receptors coupled, either directly or *via* cytosolic messengers, to the opening of ion channels in the postsynaptic membrane (Sakurai, 2007; Kukkonen and Leonard, 2014; Bear et al., 2015). However, glutamate release is typically initiated very rapidly, by one or a few presynaptic action potentials; its action is also terminated similarly promptly by a combination of extracellular diffusion and glutamate reuptake by neurons and glia, resulting in a millisecond-level on/off dynamics for ionotropic glutamate signals (Bear et al., 2015). In contrast, orexin release seems to require more prolonged presynaptic firing, and orexin-evoked postsynaptic depolarization can persist for many seconds or even minutes, presumably due to slow orexin diffusion and/or breakdown, no known reuptake mechanisms, and the long half-lives of intracellular messengers generated by orexin G-protein coupled receptors (GPCRs) (Schöne et al., 2014).

Thus, functional neural circuits in the brain can be created either by slowly-acting neurotransmitters such as neuropeptides, or by fast-acting classic neurotransmitters. In the rest of this short review, specific examples of neuropeptidergic brain circuits will be presented from the above-mentioned perspective. Our focus will be narrow and somewhat subjective, concentrating on recent insights from studies of lateral hypothalamic neuropeptidergic neurons linked to control of arousal and vigilance state switching. However, some general concepts will be proposed and experiments for probing them further will be outlined.

## Brain-Wide Projecting Peptidergic Neurons of the Non-Neuroendocrine Hypothalamus

Functionally-speaking, the hypothalamus is usually thought of as comprising two parts: the endocrine hypothalamus consisting of neurons controlling pituitary hormone release, and the non-neuroendocrine hypothalamus, which represents most of the hypothalamus in terms of volume and contains large and heterogeneous neurons that mono-synaptically innervate much of the brain (Peyron et al., 1998; Bittencourt, 2011; Bear et al., 2015).

It is the latter, non-neuroendocrine, hypothalamus that is the focus of this review. It is a complex confederation of neuronal clusters (nuclei). Most of the non-neuroendocrine hypothalamic neurons examined so far appear to synthesize and/or use classic neurotransmitters such as GABA and glutamate (Atasoy et al., 2012; Dicken et al., 2012; Schöne and Burdakov, 2012; Jego et al., 2013; Romanov et al., 2017; Mickelsen et al., 2019). In addition, most (if not all) hypothalamic neurons express a peptide neurotransmitter. Many of these neuropeptides are generally thought to be made only in the hypothalamus, for example orexin, discussed in our earlier example, is made exclusively by neurons of the lateral hypothalamus (LH) (de Lecea et al., 1998; Sakurai et al., 1998). This makes hypothalamic neuropeptidergic-producing neurons a very attractive “model system” for studying the role of neuropeptide transmission in brain-wide neural computation, brain state control, and behavior. This is because, in contrast to brain-wide synthesized transmitters such as glutamate, the origin of hypothalamus-unique neuropeptide signals is always known. This solves a major problem of interpretation in systems neuroscience, by allowing neuropeptidergic influences to be interpreted with precise knowledge of their normal origin. For example, if changes in neuronal firing in a particular brain area are observed upon exogenous application of orexin neuropeptide in brain slices, specific hypotheses can be formulated about LH interactions with these areas (Burdakov et al., 2003; van den Top et al., 2004; Sakurai, 2007; Burdakov and Gonzalez, 2009; Belle et al., 2014; Hay et al., 2014; Burdakov, 2020).

## Information Represented by LH Neuropeptidergic Neurons

Here we choose once more the LH neuropeptide orexin to exemplify some emerging concepts in neuropeptide-mediated function and neuronal connectivity, given that the LH has been long-known to be crucial for normal behavioral and brain state control. Orexin neurons play a key role in sleep/wake regulation (Inutsuka and Yamanaka, 2013) and a rather large body of evidence supports their critical role in stabilizing behavioral states (Chemelli et al., 1999; Lin et al., 1999; Hara et al., 2001; Mochizuki et al., 2004; Ma et al., 2018). Importantly, the role of orexin neurons in sleep/wake and behavioral state regulation has been shown to be primarily linked to orexin peptide release–rather than their “classic” co-transmitters (Ma et al., 2018). The peptide orexin A is itself wake promoting (Hagan et al., 1999), an effect possibly mediated *via* the histaminergic system (Huang et al., 2001).

Lateral hypothalamus lesions, either at crude anatomical level or more recently at cell-type-specific level, produce profound motor, cognitive, and sleep-wake abnormalities [reviewed in Saper et al. (2005)]. Changes in LH neuron firing are sufficient to generate diverse and profound behavioral and brain state alterations, from sleep-wake switching to specific goal-directed behaviors (Bernardis and Bellinger, 1996; Adamantidis et al., 2007; Jego et al., 2013; Mahler et al., 2014; Stuber and Wise, 2016; Blomeley et al., 2018). The firing of orexin neurons is generally higher during wake (especially active waking) and lower during sleep (Lee et al., 2005). Their stimulation increases the probability for mice to transition from sleep to wakefulness (Adamantidis et al., 2007; Carter et al., 2009), while their inhibition during the inactive (light) phase has the opposite effect in mice, promoting NREM sleep (Tsunematsu et al., 2011). Specific lesions of LH orexin neurons, such as orexin peptide knockout or orexin cell ablation, can substantially alter normal timing of vigilance state transitions in response to external context (narcolepsy) (Lin et al., 1999; Ma et al., 2018), demonstrating the critical role of orexin for moment-to-moment sensorimotor control (Chemelli et al., 1999; Hara et al., 2001; Karnani et al., 2020). This crucial role in vigilance state regulation is further supported by the fact that orexin neurons send excitatory inputs to all known wake-promoting brain regions (Scammell et al., 2017).

*In vivo*, the changes in LH orexin neuron firing rate can occur both rapidly and slowly, and alter behaviors either quickly (sub-second) or slowly (minutes to hours) (Adamantidis et al., 2007; Karnani et al., 2019). The slow changes in activity of orexin neurons are thought to represent changes in the internal body state. The LH is historically known as a glucose-sensing brain area (Oomura et al., 1969). LH glucose-sensing has more recently been mapped onto neurochemical cell types, such as orexin and melanin-concentrating hormone (MCH) neurons (Yamanaka et al., 2003; Burdakov et al., 2005; Gonzalez et al., 2008; Karnani et al., 2011; Venner et al., 2011). The LH also contains cellular and molecular sensing pathways for numerous other indicators of body state, including hormones such as leptin and ghrelin, as well as dietary amino acids (Yamanaka et al., 2003; Leinninger et al., 2009; Karnani et al., 2011; Lam et al., 2011; Burdakov et al., 2013). In addition to nutrients and hormones, orexin neurons also sense acid and CO_2_ levels, which may assist in respiratory control (Williams et al., 2007; Williams and Burdakov, 2008; Sunanaga et al., 2009). These inputs usually change LH neural firing on slow timescales, from seconds to minutes (e.g., Yamanaka et al., 2003; Williams et al., 2008). In contrast, external sensory inputs such as sound and light can alter orexin cell firing on subsecond timescales, presumably *via* direct synaptic inputs that orexin neurons receive from the rest of the brain (Mileykovskiy et al., 2005; Gonzalez et al., 2016; Karnani et al., 2020).

Lateral hypothalamus orexin cell firing may thus communicate a combined representation of fast and slow sensory variables. The consequent control of fast and slow behaviors and brain state transitions by orexin neurons has been recently reviewed elsewhere (Kosse et al., 2015; Herrera et al., 2017; Burdakov, 2019, 2020; Adamantidis et al., 2020). Below, we focus on circuit effects of endogenous orexin peptide release that may lie between orexin cell firing and behaviors or brain state transitions.

## Orexinergic vs. Glutamatergic Representations of Orexin Cell Firing in Downstream Neurons

Orexin neurons co-express several neurotransmitters in addition to orexins, such as the fast transmitter glutamate and the neuropeptide dynorphin (Chou et al., 2001; Schöne and Burdakov, 2012). Upon selective optogenetic stimulation of orexin neurons, the membrane potential responses of postsynaptic neurons have been analyzed using brain slice patch-clamp recordings in several brain regions.

**FIGURE 1:**
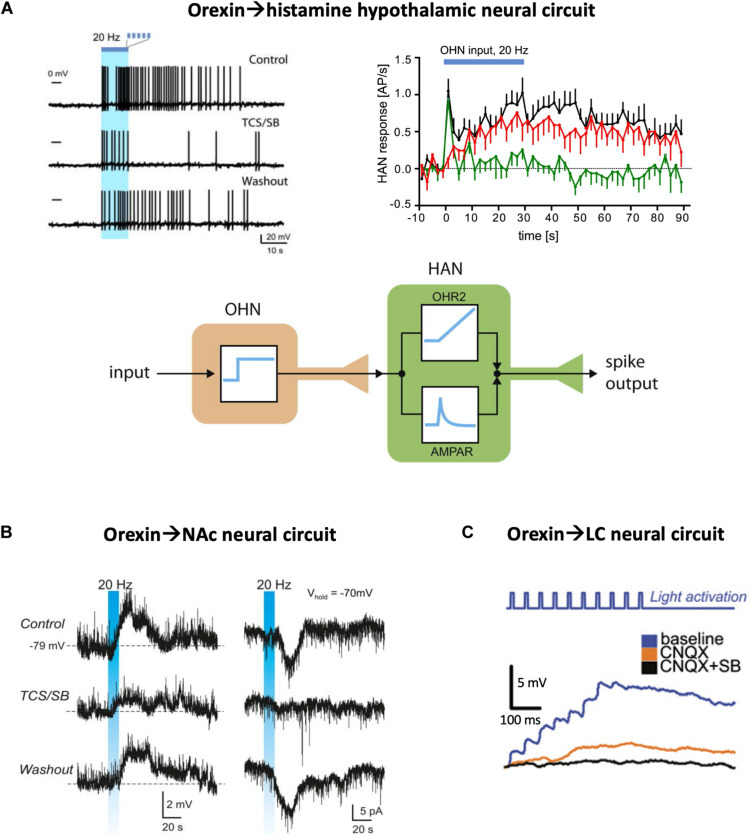
Optogenetic evidence for neuropeptide-mediated neural circuit connectivity. **(A)** Hypothalamic orexin neuron (OHN) → histamine neuron (HAN) circuit. Top left, example of HAN firing response to optogenetic stimulation of OHNs (blue bar), in the presence and absence of orexin receptor blockers (TCS/SB = OX1 and OX2 receptor antagonists SB334867 and TCS-OX2-29). Top right, the same data plotted across many trials, illustrating glutamate (green) and orexin (red) circuit connectivity (AP/s = action potentials per second). Bottom, a conceptualization of the orexin and glutamate transmission as integral-like and derivative-like parallel signals. Source: Schöne et al., 2014. **(B)** Circuit between hypothalamic orexin neurons and accumbal D2 neurons. Stimulation of orexin cell axons (blue bar) creates orexin-receptor-blockade-sensitive excitation in the postsynaptic D2 neurons (left column, membrane potential recordings; right column, membrane current recordings). Source: Blomeley et al., 2018. **(C)** Circuit between hypothalamic orexin neurons and LC noradrenaline neurons, in the presence and absence of glutamate (CNQX = AMPA receptor blocker) and orexin (SB = SB334867 orexin receptor blocker) receptor transmission. Adapted from Sears et al., 2013.

Inside the hypothalamus, the neural circuit between LH orexin and tuberomammillary histamine neurons has been examined. During constant-frequency optogenetic stimulation of orexin cells, histamine cell firing did not follow the temporal pattern of orexin cell stimulation (i.e., a square wave), but responded with a temporal dynamics reminiscent of the sum of a first order derivative and an integral of the presynaptic orexin cell activity (Schöne et al., 2014; Figure 1A). Pharmacological dissection of these responses indicated that glutamate mediated only the initial transient component of the postsynaptic response, with glutamate transmission seemingly “running out of steam” after a couple of seconds of sustained orexin cell firing. In turn, the integrative sustained component of the responses, which accounted for most downstream spikes in the orexin→histamine circuit, was mediated by orexin neuropeptide transmission, specifically by orexin type-2 GPCRs (Schöne et al., 2014). Pharmacological blockade of glutamate-driven spiking did not affect orexin-driven spiking and vice versa, suggesting that each co-transmitter acts in an isolated manner, without orexin-glutamate cross-modulation. This ability of orexin and glutamate to translate distinct features of orexin cell firing activity into sustained integrative, and transient derivative-like responses, respectively, illustrates how neuropeptides can create functional slow neural circuits that are operationally distinct from classic fast neural circuits. The integral-like nature of orexin-induced postsynaptic firing has been compared to integral feedback control signals, which are likely to be essential for stable feedback control of brain states (Kosse and Burdakov, 2014; Schone and Burdakov, 2017).

In several other hypothalamic regions, orexin-induced postsynaptic excitatory responses have also been reported, although they were created by exogenous application of orexin to brain slices rather than by endogenous orexin neurotransmission as in the above-described study (Follwell and Ferguson, 2002; Burdakov, 2004; van den Top et al., 2004; Sakurai, 2007). Interestingly, in some cases, only weak or non-existent “classic” (i.e., fast glutamatergic or GABAergic) orexin→target circuit connectivity was found in targets that display robust responses to exogenous orexin. One of such targets is LH GAD65 neurons, which are robustly excited by orexin and are required for generating normal locomotion in mice (Kosse et al., 2017). This example is interesting, because based on traditional “gold standard” connectivity mapping–i.e., simultaneous pre- and postsynaptic patch-clamp recordings or channelrhodopsin-assisted circuit mapping which focus on fast connectivity and ignore slow connectivity–it would be concluded that the orexin→GAD65 LH microcircuit does not exist (Burdakov and Karnani, 2020). Yet, when examined from a neuropeptidergic perspective, the LH GAD65 neurons display robust machinery for strong orexin→GAD65 neuropeptidergic coupling, which appears important for activation of LH GAD65 neurons *in vivo* (Kosse et al., 2017).

Outside the hypothalamus, there is also considerable evidence that neuroexcitatory orexin transmission forms functional peptidergic circuits. One example is the recently described circuit between orexin neurons and dopamine-inhibited D2 receptor-expressing medium spiny neurons of the nucleus accumbens shell (Blomeley et al., 2018). Optogenetic excitation of orexin neurons creates depolarization waves in these D2 neurons, and these waves are blocked by orexin receptor antagonists demonstrating a functional LH_orexin_→NAc_D2_ neuropeptidergic circuit (Figure 1B). This circuit is proposed to control NAc-dependent action-selection, in particular risk taking, based on orexin cell activity (Blomeley et al., 2018). In contrast to the strong effects of endogenously-released orexin, glutamatergic transmission in the same circuit appears rather weak (Blomeley et al., 2018). Similar neuropeptide-mediated circuits, albeit with a stronger glutamatergic component, have been reported between orexin neurons and locus coeruleus neurons (Sears et al., 2013; Figure 1C).

From such studies, it can be concluded that orexin neuropeptide transmission is able to create functional intra- and extra-hypothalamic neural circuits in its own right, beyond a mere modulation of fast neurotransmitters.

## How Unique Are Neuropeptides in the Slowness of Their Actions?

Describing transmitters as fast and slow is a concise way to capture their important operational characteristics. Yet, the dichotomy between fast and slow transmitters can also be viewed as somewhat artificial. Canonical “fast” transmitters, such as GABA/glutamate, also have “slow” receptors (typically GPCRs) that exert well-characterized long-lasting effects on neuronal function (Bormann, 2000; Niswender and Conn, 2010). Non-neuropeptide, intermediate-sized transmitters such as amines and acetylcholine also act on a plethora of neuronal GPCRs coupled, among other effectors, to plasmalemmal ion channels and thus to membrane excitability (Bear et al., 2015). Since non-neuropeptide transmitters are capable of slow and lasting control of neuronal excitability *via* such mechanisms, one may justifiably ask: Why does the brain need neuropeptides? What is unique about neuropeptides that other transmitters cannot achieve?

The resources invested by the brain into making neuropeptides (transcription, translation, and trafficking) are presumably not trivial. Thus, it is tempting to speculate that the 100 + neuropeptides in the brain play some unique functions, and did not evolve solely to serve as a “redundant safeguard” should something go awry with the other transmitters (or as convenient molecular markers for neuroscientists). We speculate that their unique function does not arise at the receptor level, since, as alluded above, other transmitters have similar types of receptors, namely GPCRs linked to long-lasting postsynaptic effects. It could, instead, stem from what happens to neuropeptides in the extracellular space, during the time between release and receptor binding. Unlike other neurotransmitters, which are rapidly cleared from the extracellular space by uptake into neurons and glia by specialized membrane transporters (Bear et al., 2015), neuropeptides have no known specific clearance mechanisms. Neuropeptide diffusion from their release sites will also be slower than the other transmitters, due to their larger size. While there is little quantitative information yet about how far neuropeptides spread from their natural release sites in different brain areas, and how long they stay in the extracellular space following release, we speculate that the spatiotemporal scales involved are likely to be longer than for other neurotransmitters. This may contribute to the unique reasons why neuropeptides have evolved among other transmitters. Alternatively, or in addition, these unique reasons could relate to certain advantages of neuropeptides for evolution itself. The 1 gene/1 (prepro)peptide encoding relationship of peptides vs. the enzymatic multistep synthetic pathways of other neurotransmitters might offer the evolutionary advantage of single gene duplication for generation of new neuronal identities.

## Emerging Concepts and Future Directions

In order to gain a broader understanding of functional neural circuits, we propose that neuropeptide-mediated postsynaptic signals should be routinely analyzed alongside the classic small-molecule neurotransmitters in circuit connectivity screens. While such broader analysis is becoming commonplace in functional dissections of hypothalamic circuits, it is still relatively unusual in other neural circuits, e.g., in cerebral cortex where neuropeptides such as NPY and somatostatin are also abundantly expressed (Karnani et al., 2016). Thanks to the current genetic tools, studies are able to focus on neuronal populations that express a given neuropeptide. And yet, in most cases, the exact role of the neuropeptidergic release vs. that of the co-released classic neurotransmitter(s) is rarely unraveled. For example, the role of galaninergic neurons in sleep regulation has been rather extensively studied, in particular the sleep-promoting role of galanin-expressing GABAergic neurons of the ventrolateral preoptic area (Steiger and Holsboer, 1997; Sherin et al., 1998). A more recent study also showed that galaninergic neurons of the dorsomedial hypothalamus can be divided into two distinctly-projecting subsets: one suppressed during REM sleep and whose activation promotes NREM sleep and opposes REM sleep, the other with exact opposite patterns and effects (Chen et al., 2018). But these studies fail to clarify which of GABA or galanin–that these neurons co-express–mediates the reported effect on sleep regulation (Sherin et al., 1998; Chen et al., 2018). Thus, expanding circuit connectivity screens to neuropeptides and their specific actions may shed light on the mechanisms through which neural circuits solve the challenging task of exerting stable control over the brain and body in a rapidly changing world (Kosse and Burdakov, 2014).

Several emerging features of peptidergic neurotransmission should be kept in mind while probing neuropeptidergic connectivity of a neural circuit. First, there may be a presynaptic frequency threshold for neuropeptide release that is higher than that for small transmitters such as GABA or glutamate (Verhage et al., 1991; Schöne et al., 2014). It is therefore important to screen a range of presynaptic frequencies. Second, neuropeptide release and/or action seems to build-up slowly during steady presynaptic stimulation, and also decays slowly, sometimes over many seconds, rather than a few milliseconds as in the case of glutamate or GABA fast transmission (Schöne et al., 2014; Blomeley et al., 2018). It is therefore important to screen the effects of prolonged presynaptic firing trains (especially where prolonged firing is normally displayed by the presynaptic neurons *in vivo*), and to allow sufficient time for the postsynaptic response to appear. Third, while the above-chosen example of orexin illustrates how it directly creates postsynaptic excitation, this is not the case of all neuropeptides. Even within the LH, the MCH neuropeptide, which is made by neurons intermixed with (but distinct from) orexin neurons, does not appear to have direct effects on postsynaptic membrane potential but rather seems to act by altering GABA or glutamate signaling, which could supply a lasting permissive signal [akin to an eligibility trace (Gerstner et al., 2018)] for creating or erasing certain kinds of memory (Adamantidis and de Lecea, 2009; Izawa et al., 2019; Kosse and Burdakov, 2019; Burdakov and Peleg-Raibstein, 2020; Concetti et al., 2020). Thus, “modulatory” actions of neuropeptides should continue to be examined, even though some neuropeptides such as orexin do not require them to form functional neural circuits.

In summary, the ability of neuropeptidergic postsynaptic effects to substantially outlast presynaptic firing may bind together fast and slow brain functions in a way that cannot be achieved by fast transmitters alone. Slow on/off neuropeptide signals may thus enable core brain functions, such as creating temporal eligibility traces for memory formation and information routing. Neuropeptides that directly affect the firing of postsynaptic neurons can, in addition, create functional neural circuits able to perform control-relevant computations such as signal integration. This gives peptidergic neural circuits unique advantages for efficient neuronal processing and feedback control of consciousness.

## Author Contributions

DB and MG wrote this review. Both authors contributed to the article and approved the submitted version.

## Conflict of Interest

The authors declare that the research was conducted in the absence of any commercial or financial relationships that could be construed as a potential conflict of interest. The reviewer WW declared a past co-authorship with one of the authors, DB, to the handling editor.
